# Genetic Algorithm-Based Human Mental Stress Detection and Alerting in Internet of Things

**DOI:** 10.1155/2022/4086213

**Published:** 2022-08-31

**Authors:** Hatem S. A. Hamatta, Kakoli Banerjee, Harishchander Anandaram, Mohammad Shabbir Alam, C. Anand Deva Durai, B. Parvathi Devi, Hemant Palivela, R. Rajagopal, Alazar Yeshitla

**Affiliations:** ^1^Department of Applied Sciences, Aqaba University College, Al Balqa Applied University, Aqaba, Jordan; ^2^Department of Computer Science and Engineering, JSS Academy of Technical Education, Noida, Uttar Pradesh, India; ^3^Centre for Excellence in Computational Engineering and Networking, Amrita Vishwa Vidyapeetham, Coimbatore, Tamil Nadu, India; ^4^Department of Computer Science, Jazan University, Jizan, Saudi Arabia; ^5^College of Computer Science, King Khalid University, Abha, Saudi Arabia; ^6^Department of Computer Science, Sri Sarada College for Women (Autonomous), Affiliated to Manonmaniam Sundaranar University, Tirunelveli, Tamil Nadu, India; ^7^Accenture, MDC5A Building Number 2, MIDC INDL Area, Airoli, Navi Mumbai, Maharashtra, India; ^8^Department of Computer Science and Engineering, Narsimha Reddy Engineering College, Hyderabad, Telangana, India; ^9^Department of Biotechnology, College of Biological and Chemical Engineering, Addis Ababa Science and Technology University, Addis Ababa, Ethiopia

## Abstract

Healthcare is one of the emerging application fields in the Internet of Things (IoT). Stress is a heightened psycho-physiological condition of the human that occurs in response to major objects or events. Stress factors are environmental elements that lead to stress. A person's emotional well-being can be negatively impacted by long-term exposure to several stresses affecting at the same time, which can cause chronic health issues. To avoid strain problems, it is vital to recognize them in their early stages, which can only be done through regular stress monitoring. Wearable gadgets offer constant and real information collecting, which aids in experiencing an increase. An investigation of stress discovery using detecting devices and deep learning-based is implemented in this work. This proposed work investigates stress detection techniques that are utilized with detecting hardware, for example, electroencephalography (EEG), photoplethysmography (PPG), and the Galvanic skin reaction (GSR) as well as in various conditions including traveling and learning. A genetic algorithm is utilized to separate the features, and the ECNN-LSTM is utilized to classify the given information by utilizing the DEAP dataset. Before that, preprocessing strategies are proposed for eliminating artifacts in the signal. Then, the stress that is beyond the threshold value is reached the emergency/alert state; in that case, an expert who predicts the mental stress sends the report to the patient/doctor through the Internet. Finally, the performance is evaluated and compared with the traditional approaches in terms of accuracy, f1-score, precision, and recall.

## 1. Introduction

Mental stress is one of the most dangerous diseases nowadays, it is caused by many psychological problems, general stress is common for all types of the human being, and it occurs in the routine life of human beings, but prolonged stress causes more affection in the human body; sometimes, it causes the chronic diseases, and sometimes, it may occur in the genetic disorder.

The stress level in a human being has been detected by using EEG, ERP, EDA, and the HR device, and this is all above the sensor device which is used to evaluate the mental stress in the human body.

The multilevel hybrid model is used to diagnose the multiple signals of mental stress such as sad, fear, and the angry [1]. The Arduino microcontroller is one of the devices that are used to measure the heart rate value instead of human stress and the normal stage of the body conditions. Thus, it gives better accuracy of stress level when compared to the other technique [2].

In many cases, the mental stress leads to the most vulnerable diseases in human beings; thus, the identification of the diseases is might more important, the researchers move on the side of the Internet of Things basis sensor device, and this type of device provides better accuracy in both the machine learning and the deep learning algorithm [3]. The early detection and the prevention of mental stress may be more crucial.

### 1.1. Contribution

The main contribution of this work is to detect human mental stress using the genetic-featured algorithm on the basis of the Internet of things, and mostly, artificial intelligence is utilized in the Internet of things which helps to identify the accuracy and the classification results of the mental stress.

### 1.2. Objective

The proposed work uses a convolutional neural network for classifying the given input image since CNN is one of the most important techniques in image recognition because it evaluated each and every pixel in the given data. The genetic algorithm is used to extract the feature in the evaluation of the brain tumor.

## 2. Review of Literature

Parab et al. [4] implemented the stress and the emotion analysis using IoT and deep learning, the identification of the electro-dermal activity, and emotional and sentimental analysis using the amalgamation concept. Mainly, this concept identified the stress level on the basis of the various state of mind and introduced various identifications such as emotional sensation and speech recognition using advanced technology using IoT and the deep neural network.

Mozafari et al. [5] implemented the IoT-enabled multimodal mental stress monitoring, the detection of the human stress level using artificial intelligence (AI), and the SVM algorithm, the feature is extracted by using the fisher method, and the classification technique handles with/without PCA method. By using this method, it detected the multimodal emotions and their classification based on the accuracy level.

Verma and Sood [6] implemented a comprehensive framework for tracking student stress in an IoT environment of fog clouds. The Bayesian belief network classifies the input data into normal and abnormal categories based on their analysis, then inserts the student data into two stages of a temporal dynamic Bayesian network (TDBN). This predictive model contains four parameters for further analysis such as leaf node evidence, context, student health trait, and the workload context; it helped to analyze the accurate level of emotions in the human being.

Padmaja et al. [7] implemented the IoT-based stress detection and health monitoring system, the detection of the human stress level wherever it can detect by using many types of equipment, but the accurate predictions are more challenging one in now a day; sometimes, the patient looks healthier, but they are also suffered from stress, and this problem should be considered as the chronic illness. Therefore, the IoT things introduced many sensor devices helped to detect the accurate value of the stress level.

Rachakonda et al. [8] implemented the deep neural network with dataset collection of three waveforms such as 2000, 4000, and 6000 and conducting the specific samples. It provides the testing and the training dataset evaluation. The DNN-integrated edge device was implemented for stress level detection in the IoMT for Stress-Lysis. Therefore, the final results showed the enhanced accuracy and the better classification results.

Uday et al. [9] implemented the detection of stress using wearable sensors in the IoT platform, the sensor devices such as HRV, galvanic skin response, and the electro-dermal activity help to detect the various form of the stress evaluation in the human body, and this paper introduced the MATLAB visualization for further setup of the specification in the Internet of things. This helped to detect the better accuracy and the perfect detection of the emotional and the stress calculation for the human being.

Raval [10] implemented stress detection using a convolutional neural network and the Internet of things. Earlier, artificial intelligence in the Internet of things helps to identify the better accuracy and the classification of emotional classification and stress identification. Thus, it implemented the CNN for better image classification that CNN produces better classification results when compared to the other technique.

Shon et al. [11] implemented the emotional stress state detection using genetic algorithm-based feature selection on EEG signals, the genetic algorithm for the feature extraction, and the K-nearest neighbor used to classify the given dataset by using the DEAP dataset with a public collection of EEG signals.

Das Chakladar et al. [12] implemented the EEG-based mental workload estimation using a deep BLSTM-LSTM network and evolutionary algorithm. The ERP and the EEG are the levels of signal measurement which are used to calculate the mental stress; thus, this paper implements the multilevel alignment to detect the accuracy value in the given input data. This paper implements the STEW dataset, this paper implements the grey wolf optimized technique, and it provides better optimization when compared to the GA and the PSO feature extraction technique. The bidirectional long short-term memory and the long short-term memory are used to evaluate the mental stress using a multilevel hybrid algorithm.

Santhosh Kumar et al. [13] implemented the fast time scale genetic algorithm-based image segmentation using a cellular neural network (CNN), and the genetic algorithm and the CNN technique are considered the advanced technology in brain stress evaluation. The main advantage of the genetic algorithm is to predict and covert the image into a binary value, and the CNN is used to detect the image recognition using pixel value.

## 3. Overview of the Proposed Approach

The overview of the proposed approach implies that input image, preprocessing technique, segmentation, and feature extraction, and finally, the classification process takes place.

**Figure 1:**
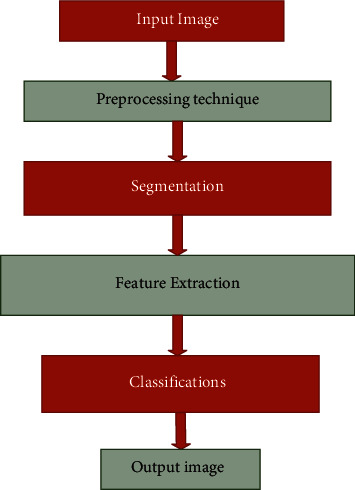
Overview of the proposed approach.

Figure 1 implies the overview of the proposed approach, and in the very first stage, the collected dataset is inserted into the input image. This image goes through the preprocessing technique, and this technique helps to reduce the noise in the filter and improve the image quality. The main function of the preprocessing technique helps to convert the image into RGB to grey scale image. The segmentation technique is nothing, but the large amounts of data are split up into the small amount of data, to further classify the accuracy process. The feature extraction is the part of the dimensional reduction and smoothening of the images and also helps to sharpen the edges. The main function of feature extraction is to divide the input data into more useable groups, and it helps to provide better classification in image processing. Then, the classification technique is used to classify the image on the basis of the stress level in the human body. This research uses three sensors to classify the different types of sensor devices using different classification schemes. In some cases, the comparison of the algorithms is better to detect the accuracy.

## 4. Proposed Approach

**Figure 2:**
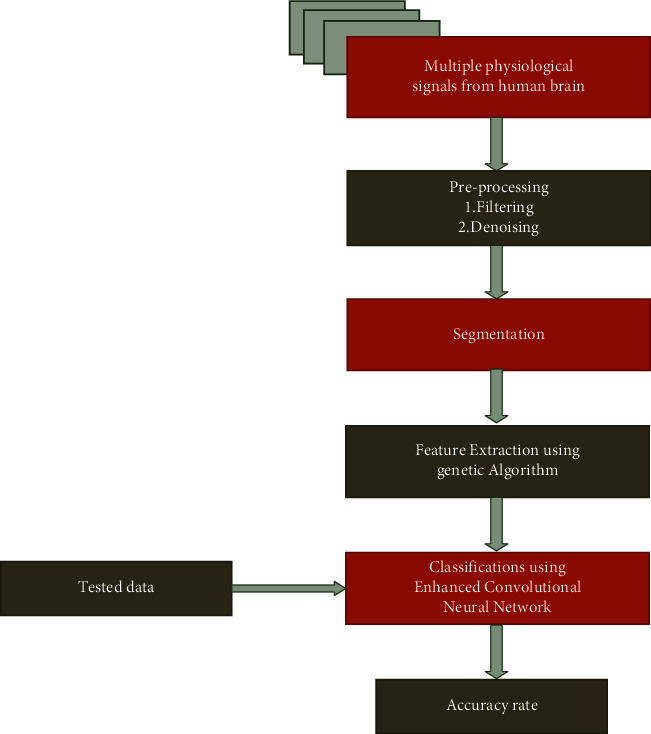
Proposed approach.

Figure 2 implements the proposed approach for the mental stress detection and the classification technique using a deep learning network and the preprocessing technique are gathered by using the filter and the denoising technique. The segmentation technique is used to split up the huge amount of input data into further organized groups; after the completion of the segmentation technique, the feature extraction technique is handled by using the genetic algorithm. The genetic algorithm provides better accuracy for reducing the dimensionality reduction in the image when compared to the PCA or any other existing technique [1–3, 14].

### 4.1. Datasets

In our paper, the DEAP dataset is implemented [14–19]. For collecting the various types of physiological signals using EEG, PPG, and GSR sensor devices, the public dataset is used for the function of detection of mental stress using EEG, PPG, and GSR devices. Thus, the 32 healthy participate samples are given as the input for our mental stress detection. They are all hearing 40 types of video songs at this time. The level of mental stress and the calm level can be detected by using three types of sensors that are implemented in this paper namely EEG, PPG, and the GSR [14, 20–29].

The EEG is nothing but the brain activity that can be detected by using cost-effective methods that are considered such types of the EEG signal device; otherwise, it is called one of the brain-activity-detecting electrodes. The PPG is considered one of the emotional recognition signal devices which are used to evaluate the human present and past state comparison in the form of human biological activities. The Galvanic skin response is nothing, but the GSR is considered as the epidermis conductance that helps to the evaluation stress level of the human body.

### 4.2. Preprocessing Technique

The preprocessing technique is used to detect the error in the image by adding some filter, and in this paper, the high pass filter is implemented to remove the error or attack effect in the form of the power line interference in the sensor devices such as EEG, PPG, and the GSR. Also, the low pass filter is used to remove the Gaussian noise in the given input image. The high pass filter is used at the frequency of 0.5 Hz, and the low pass filter is used at the frequency of 500 Hz. The butter-worth filter is used to remove the reverse forwarding action in the given data samples.

(1) $$\begin{array}{c} H\left(t,u\right)=\frac{1}{D\left(t,u\right)/D_{u}}. \end{array}$$

The high pass filter is used to detect the sharpening of and smoothening of the edges in the given data sample. It gives the brightness for the connection area of the images. The low pass filter also helps to increase the smoothening in the given image and also enhances the image equality by enhancing the adjacent pixel values. The main function of the low pass filter helps to reduce the spatial noise in the image. Denoise is nothing but removing the noise in the image or any other signal. This is one of the major challenges in the dataset.

The arousal and the valence space are one of the important techniques to predict the signal alignments in the EEG, PPG, and the GSR.

### 4.3. Segmentation

One of the most important techniques is image segmentation, which divides an image's pixels into numerous groups that have significance and facilitates classification and easy extraction. The main function of the segmentation process helps to reduce the complexity of the image [17]. This paper implements that windows 1s and 4s are used to segment the arithmetic task for the mental stress image.

### 4.4. Genetic Algorithm

The genetic algorithm in the mental stress plays a vital role, and in some cases, the prolonged mental stress is caused by genetic disorders, and the normally genetic algorithm provides a better extraction of the given data and reduces the dimensional quality of the image.

**Figure 3:**
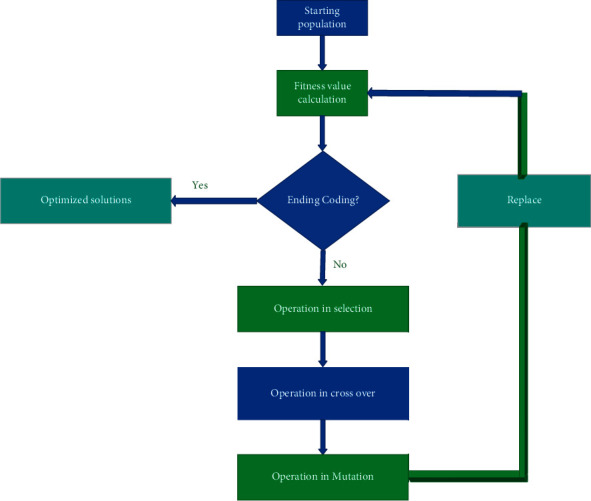
Genetic algorithm.

Figure 3 represents the genetic algorithm. It represents the basic concepts of the genetic algorithm [18].

The GA consists of the major operations namely selection crossover and the mutations technique. The input dataset is considerable with this algorithm with the help of the sensor devices namely EEG, PPG, and the GSR that show the exact dimensional reduction value to the classifier. Thus, it provides a better-enhanced result of extraction when compared to the other technique [14].

### 4.5. Enhanced Convolutional Neural Network-LSTM

The enhanced convolutional neural network with an LSTM classifier was used to classify the given mental stress image based on the dataset. The EEG, PPG, and the GSK are used to collect the data from the human being.

**Figure 4:**
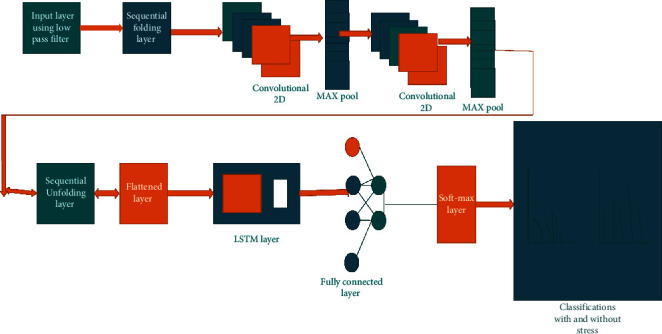
ECNN-LSTM.

Figure 4 compared to the current method, the ECNN-LSTM classifier provides greater accuracy, specificity, and sensitivity when used to classify the input. The given input data are convolute to remove noise, and then, this image goes through the max pooling. The max pooling helps to reduce the over-fitting in the image. The long short-term memory network is considered as the noise removal technique, and it helps to remove the spatial noise in the given dataset [19].

(2) $$\begin{array}{c} \text{ACCURACY}=\frac{\text{RP}+\text{RN}}{\text{RP}+\text{RN}+\text{FP}+\text{FN}},\\ \text{SENSITIVITY}=\frac{\text{FP}}{\text{RP}+\text{RN}},\\ \text{PRECISION}=\frac{\text{RP}}{\text{RP}+\text{RN}},\\ \text{NEGATIVE PREDICTIVE VALUE}=\frac{\text{RN}}{\text{RN}+\text{FN}}. \end{array}$$

The above equation shows the accuracy, sensitivity, precision, and the negative predictive value based on the ECNN-LSTM classifier.

## 5. Comparison Analysis

When compared to the current method, the method in our study offers 99.9% accuracy. The existing technique contains a support vector machine, random forest method, KNN, cubic SVM, and the CNN. The graph illustrates an overall comparison of the results based on classification accuracy, sensitivity, and precision values [14]. When compared to the current technique, the implementation in our research produces improved output.

**Figure 5:**
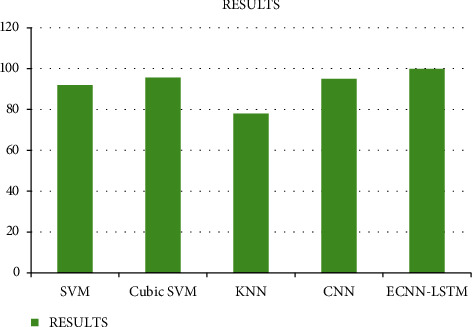
Results.

Figure 5 shows the results for the comparison analysis based on the classification accuracy.

## 6. Conclusion and Future Work

A detailed study of stress detection utilizing sensing devices and associated deep learning-based is offered in this paper. This research looks into stress detection methods that are used with sensing equipment such as electroencephalography (EEG), photoplethysmography (PPG), and the Galvanic skin response (GSR) as well as in different environments including traveling and learning. A genetic algorithm is used to extract the features, and this paper implements the ECNN-LSTM classifications technique results that show the perfect accuracy of the given input data, which helps to predict the calm and the stress stage of the human being based on their emotion, and it was calculated by using EEG, GSR, and the PPG sensor devices.

Our future work is based on the comparison of the enhanced technique in the SVM and the ECNN-LSTM with MTH feature extraction, to predict the classification accuracy.

## Data Availability

The datasets used and/or analyzed during the current study are available from the corresponding author on reasonable request.
